# Effect of Anderson localization on light emission from gold nanoparticle aggregates

**DOI:** 10.3762/bjnano.7.192

**Published:** 2016-12-16

**Authors:** Mohamed H Abdellatif, Marco Salerno, Gaser N Abdelrasoul, Ioannis Liakos, Alice Scarpellini, Sergio Marras, Alberto Diaspro

**Affiliations:** 1 Nanophysics Department, Istituto Italiano di Tecnologia, via Morego, 30, I-16163 Genova, Italy; 2 Smart Materials Group, Istituto Italiano di Tecnologia, via Morego, 30, I-16163 Genova, Italy; 3 Nanochemistry Department, Istituto Italiano di Tecnologia, via Morego, 30, I-16163 Genova, Italy; 4 Department of Physics, University of Genoa, via Dodecaneso, 33, I-16146 Genova, Italy

**Keywords:** Anderson localization, gold nanoparticle aggregates, photoluminescence, plasmons, surface plasmon resonance

## Abstract

The localization of light known as Anderson localization is a common phenomenon characterizing aggregates of metallic nanostructures. The electromagnetic energy of visible light can be localized inside nanostructures below the diffraction limit by converting the optical modes into nonradiative surface plasmon resonances. The energy of the confined photons is correlated to the size and shape of the nanostructured system. In this work, we studied the photoluminescence dependence of aggregates of 14 nm diameter gold nanoparticles (AuNPs) synthesized by drop-casting a liquid suspension on two different substrates of glass and quartz. The AuNP aggregates were characterized by electron microscopy, X-ray diffraction and X-ray photoelectron spectroscopy. The dielectric constant of the surrounding medium plays a crucial role in determining the aggregate geometry, which affects the Anderson localization of light in the aggregates and hence causes a red-shift in the plasmonic resonance and in the photoluminescence emission. The geometry of the gold nanoparticle aggregates determine the strength of the Anderson localization, and hence, the light emission from the aggregates. The photoluminescence lifetime was found to be dependent on the AuNP aggregate geometry and the dielectric constant of the medium.

## Introduction

The process of localization of waves has been observed in several physical phenomena, such as for excitons in semiconductor nanostructures [1] and for surface plasmon polaritons at the interface between metallic and dielectric films [2–3]. Light trapping in amorphous aggregates of metal nanoparticles known as Anderson localization [3–4] can lead to pronounced optoelectronic effects. The photon interaction with a dense collection of states can lead to coherent scattering [5] and pronounced spatial fluctuations in the local density of states, where the wavefunction of the optical mode is localized and exhibits a fractal shape [6–8]. The localized modes have energy 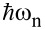, which present a highly sensitive dependence on the dimensionality and geometry of the nanostructured system. Their electric field at position ***r*** and time *t* can be described by the following equation:

[1]
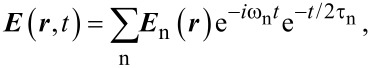


where ***E***_n_(***r***) is the local electric field at position ***r*** for each individual eigenmode n and ω_n_ and τ_n_ are the characteristic angular frequency and time constant of the eigenmode, respectively. The photon localization phenomena have been used intensively in optical antennas [9]. The understanding of the behavior of optical confinement would help the growing areas of photodetection [10], light emission [11], sensing [12] and spectroscopy [1].

Another crucial property of metals is the strong optical nonlinearity, which brings in many opportunities for useful applications. For example, the third order nonlinear susceptibility χ^(3)^ of gold nanoparticles (AuNPs) (χ^(3)^ < 1 nm^2^V^−2^) [13] is three orders of magnitude higher than that of nonlinear crystals such as potassium di-hydrogen phosphate, potassium titanyl phosphate or lithium niobate. This effect can be ascribed to the fractal-like shape or statistical self-similarity of the AuNP arrangement [14]. The concept of fractal-like design in optics was introduced for the first time by Stockman [14]. In a chain of particles, the self-similar pattern allowed nanofocusing and high field enhancement to be achieved in the subwavelength regime. The principle of confinement of optical pulses in metal nanoparticles arises from the existence of plasmons in metals, consisting of collective oscillations of an electron gas.

In this work, we investigated the dependence of photoluminescence (PL) on the aggregation of AuNPs ranging from primary colloidal AuNPs to AuNP aggregates resulting from deposition on two different dielectric substrates of glass and quartz. The rough surface of the aggregated AuNPs results in Anderson localization of different degrees.

## Results and Discussion

The X-ray spectra and TEM images of the primary AuNP crystals are presented in our previous work [8] where the same nanoparticles were used. Four peaks are resolved and were assigned to the diffraction planes (111), (200), (220), and (311). The analysis of these spectra revealed a primary particle size of ≈14 nm.

For spherical nanoparticles, the condition of plasmon resonance excitation is satisfied when ε_real_ = −2ε_m_, where ε_real_ is the real part of the dielectric constant of the particle material and ε_m_ is the dielectric constant of the medium. For nonspherical nanoparticles, the electron oscillation is nonisotropic and localized along the principal axis [15] or at the points of maximum surface curvature. The asymmetry in localization then gives rise to additional shape-dependent depolarization of the plasmon, which results in the splitting of the plasmonic resonance into several modes. According to the Drude free-electron model [16], the electron resonance for small spherical metallic nanoparticles is described according to the following expression for the static polarizability α:

[2]
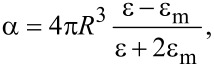


where *R* is the particle radius, ε is the complex dielectric constant of the nanoparticle metal, and ε_m_ is the dielectric constant of the medium. However, the plasmon frequency of the arbitrarily shaped particle can be determined by solving the Maxwell equations for any arbitrarily complex nanostructure geometry, as in the case of the AuNP aggregates investigated here.

**Figure 1:**
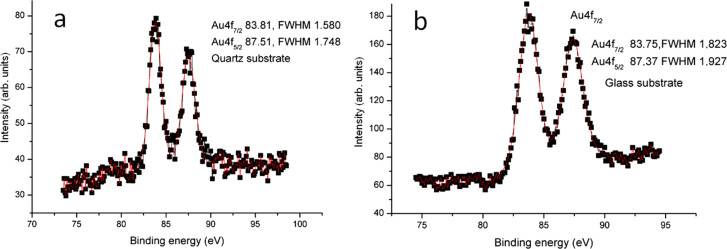
XPS spectra for the AuNPs drop-cast on a) quartz and b) glass.

In Figure 1a,b the XPS spectra of AuNPs drop-cast on glass and quartz substrates are shown. The values of the binding energy are also reported. The data shows that the surface state of the AuNPs is different between the two systems of AuNPs/quartz and AuNPs/glass. The binding energies are higher on quartz, with a difference for the 4f_7/2_ core level of 0.06 eV, and for the core level 4f_5/2_ of 0.14 eV. These differences are attributed to the different coordination number induced by the geometrical factors of the aggregates [17], i.e., their likely different aggregation density. The geometrical factors determine the extent of Anderson localization in the aggregates since the electromagnetic interaction between radiation and metal is limited by the geometry of the AuNP aggregates, which appears as a delay between the driving field and the electrical response. As a consequence of this effect, the electrons in the AuNP aggregates respond to an effective wavelength λ_eff_ [18] rather than the wavelength λ of the incident radiation, according to:

[3]
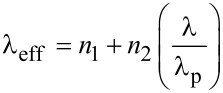


where λ_p_ is the plasmon wavelength, and *n*_1_ and *n*_2_ are proper geometrical constants also depending upon static dielectric properties [18]. Since light localization is due to the optical coupling of the visible electromagnetic radiation with the plasmon oscillation, this quantized plasma oscillation can propagate along the metal depending on the shape and size of the AuNP aggregates.

The modulation of the plasmonic resonance to the inter-subband electron transition results in hybridization of the orbitals in the AuNPs. The plasmon hybridization theory [19] is used to predict the atomic orbital interactions. The hybridized plasmon produced here results in the splitting of the plasmonic resonance into a higher energy antisymmetric plasmon mode and a lower energy symmetric mode. The symmetric plasmon has a larger dipole moment and couples easily with light giving rise to plasmon absorption [20]. This can be seen in the PL emission from the AuNPs, since field enhancement due to confinement is possible by controlling the gaps and distances between the nanoparticles to create hot spots. The simplest system of generation of such hot spots is through aggregation of nanoparticles, as seen in the work of Anker et al. [12]. The aggregate mass, *m*, which affects the plasmonic absorption can be introduced in the calculation using the Brownian aggregation rate, according to [21–22]. As the particles aggregate, the aggregate mass *m* increases with the aggregate radius *r* according to *r^D^*, where *D* is the fractal dimension, which describes the complexity of the fractal object [7]. The interaction of two aggregates [23–24] of mass *m* and *m*’ can be described in terms of a kinetic parameter *K*_B_ defined as follows [25]:

[4]
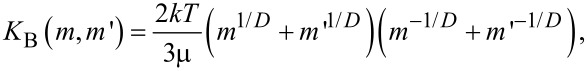


where μ is the viscosity of the medium, *k* is the Boltzmann constant, and *T* is the absolute temperature. *K*_B_ describes the Brownian aggregation rate at temperature *T* as a function of both *m* and *D,* the fractal dimension of the aggregate. Accordingly, the shift in spectral absorption due to the change in plasmon resonance upon aggregation can be similarly described [5–6], along with the resulting modifications in the optical properties [26].

**Figure 2:**
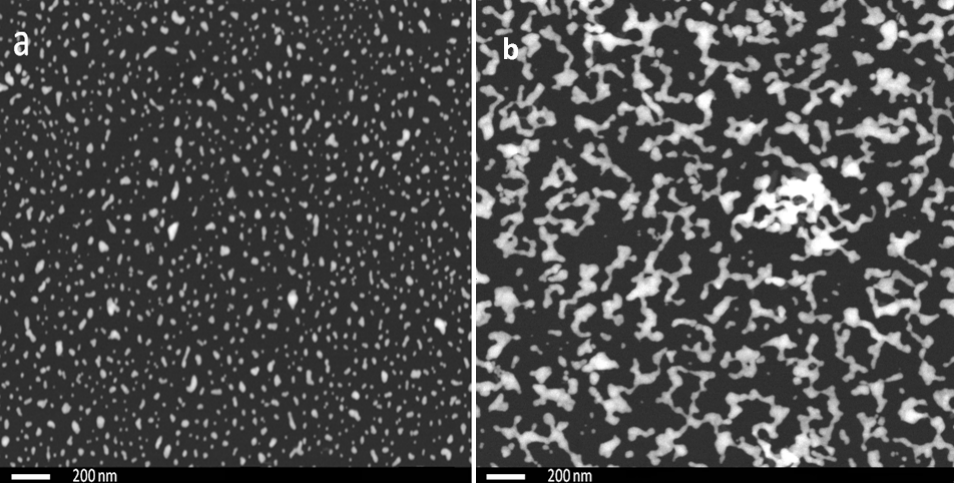
SEM images (50,000× magnification) of AuNPs on different substrates of a) glass, b) quartz.

Figure 2a,b shows the SEM images of AuNPs deposited on glass and quartz substrates, respectively. The AuNPs on the glass substrate wet the whole area of the glass with a low degree of aggregation while on the quartz substrate the AuNPs are more aggregated, which is expected due to the influence the optical properties.

The different aggregation behavior could be due to possible differences in the substrate morphology. Actually, the importance of both texture and above all the roughness of the material surfaces for many disparate physical/chemical properties are well-known and documented throughout the existing scientific literature [27–30]. In fact, we acquired atomic force microscopy images (Supporting Information File 1 Figure S1) that demonstrate the equally flat and smooth background of both the quartz and glass substrates used (RMS roughness of 0.20 and 0.15 nm on 5 × 5 µm^2^ scan areas, respectively), without a statistically significant difference, and this can rule out the possible effects due to substrate morphology.

To understand the effect of aggregation on the optical properties we apply the model introduced by Smith et al. [31], in which the probability of aggregation of a pair particles *K*(*r*,*r*’) (with radius *r* and *r*’), shows the following dependence [25]:

[5]
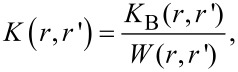


where *K*_B_(*r*,*r*’) is the same Brownian aggregation rate as in Equation 4 yet expressed here in terms of the particle positions and sizes (basically their geometry), and *W*(*r*,*r*’) is the stability ratio. The effect of interparticle interaction on the aggregation rate results into the following expression [14–15]:

[6]
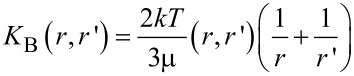


The stability ratio can be expressed as:

[7]
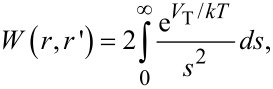


where *s* is the interparticle distance and *V*_T_ is the total interaction potential, which according to the classical model of DLVO theory [32–33] is made of is two parts [25]:

[8]
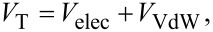


where *V*_elec_ is the electrostatic repulsion potential due to Coulomb force, and *V*_VdW_ is the Van der Waals interaction potential. The electrostatic repulsion can be calculated depending on the value of κα, the product between the inverse Debye length κ, and the particle radius α.

**Figure 3:**
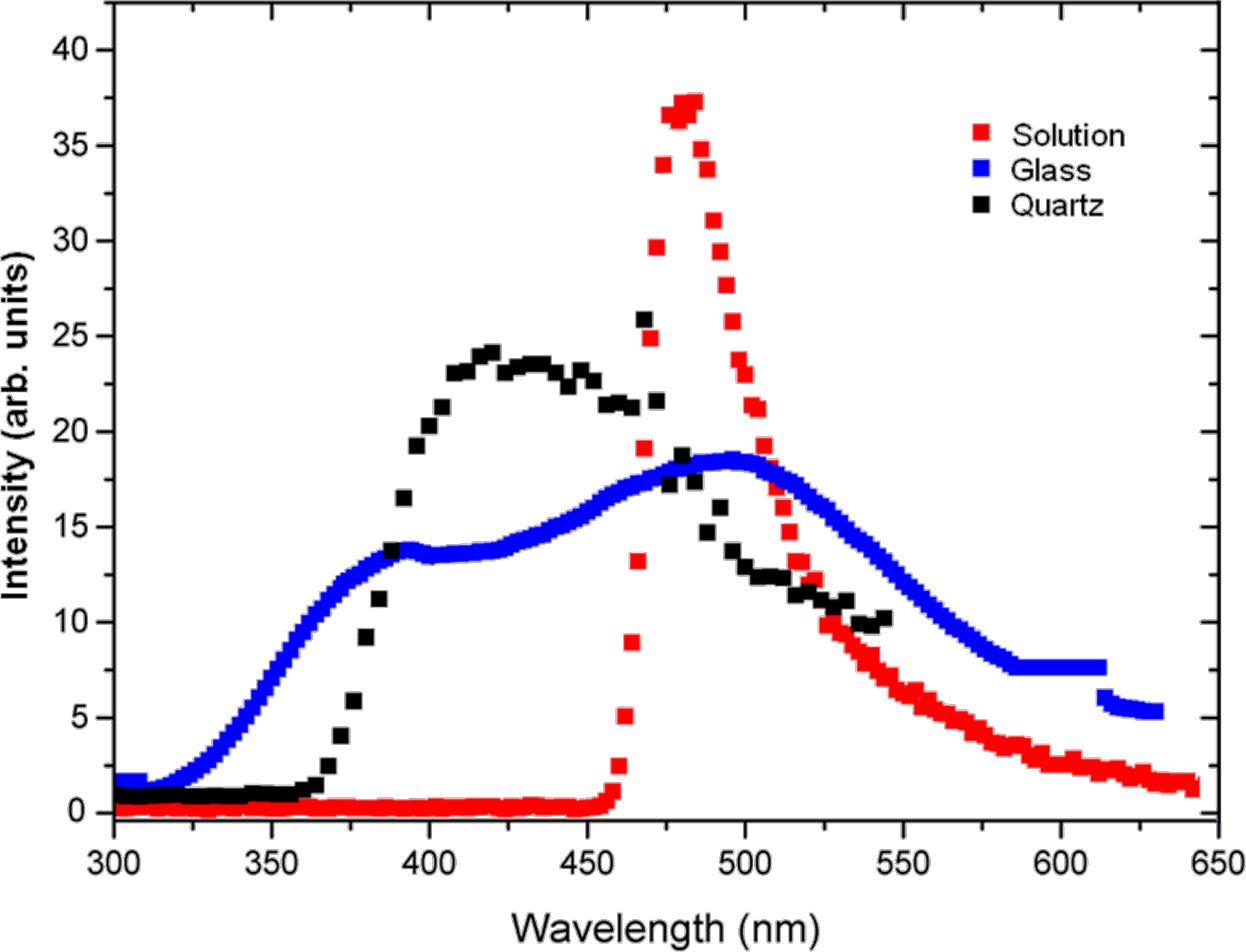
PL of AuNPs drop cast on a glass substrate (blue data points) and, on a quartz substrate (black data points). The profile of PL emission from the pristine AuNPs in aqueous solution is also shown for comparison (red data points).

The stability ratio was calculated theoretically for AuNPs on the glass and on quartz and found to be around 10^3^ and 10^5^, respectively. The stability ratio increases if the aggregate size increases, which often leads to fractal formation [34]. The change in optical properties of the AuNPs is attributed to the difference in aggregation that depends mainly on the dielectric constant of the surrounding materials, following the Drude model [16,35], and on the dielectric constant of the particles. We studied the UV–vis absorption of the AuNPs in water solution as a reference sample, assuming no aggregation in the colloidal solution. When another two samples were drop cast on the glass and on the quartz substrates, the difference in medium dielectric constant induced a different aggregation pattern according to the Derjaguin–Landau–Verwey–Overbeek (DLVO) theory [32–33], which in turn induced different strengths of plasmonic hotspots. Figure 3 depicts the PL emission from AuNPs dispersed in water solution, on a glass and on a quartz substrate, after excitation at 300 nm wavelength. Figure 2a shows that the samples differ only in the aggregation pattern and mass, in which the coordination number and geometry are supposed to be the main difference, which is confirmed by the shift in the XPS data in Figure 1.

**Figure 4:**
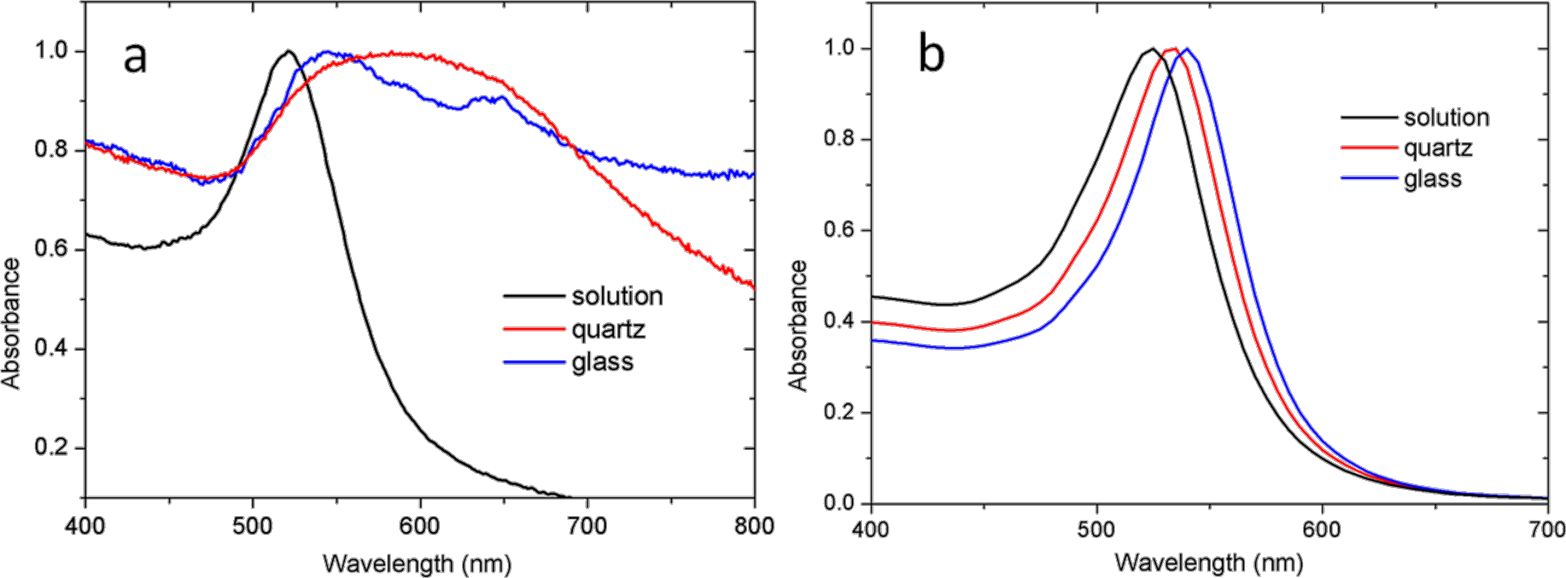
Absorption spectra of AuNPs, either in solution (black line) or drop cast on glass (red line) or on quartz (blue line). a) measured spectra, b) theoretical modeling for 14 nm diameter spherical nanoparticles in the different medium.

**Figure 5:**
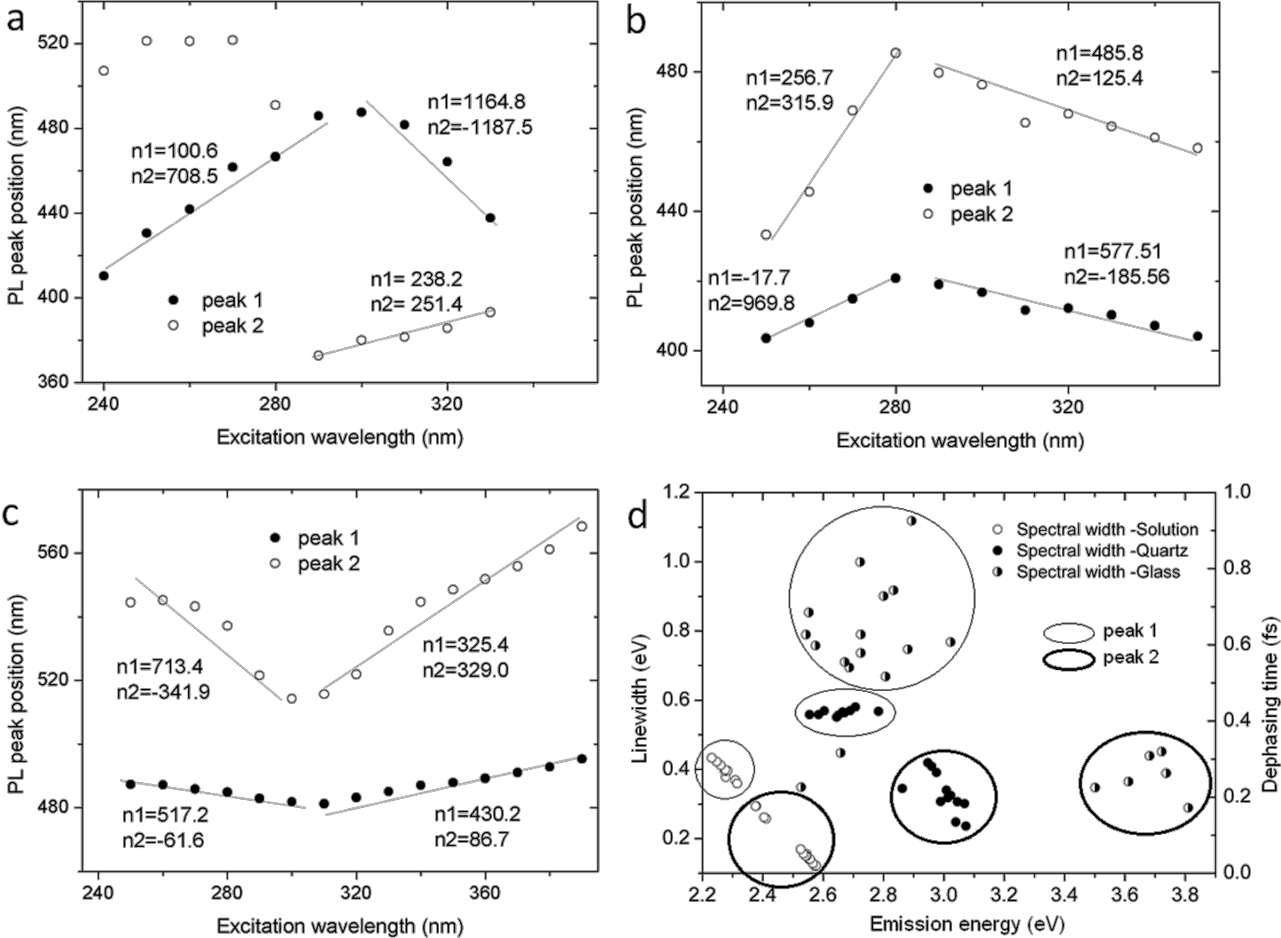
The shift of the PL peaks with changing excitation wavelength is shown for AuNPs on a) glass and b) quartz substrate and c) in solution. The red line represents the theoretical fitting. d) is the dephasing time for AuNPs in solution, deposited on quartz, and deposited on glass.

In Figure 3, the PL spectra of the AuNPs in all three forms (in solution and drop-cast on glass or quartz) are presented. It can be observed that the emission peaks are narrower in the case of the quartz substrate, and a splitting of the peak occurs (into so-called peak 1 and peak 2) that is more pronounced in the case of the glass substrate. As mentioned above, the splitting of the emission peak is due to the splitting in the geometrically dependent hybridized particle plasmon resonance [36], which can be interpreted by treating the plasmon as a quasi-particle that decays by PL radiation. The PL peak positions undergo a red-shift (shown later in Figure 5) that is due to the higher aggregation mass of AuNPs on the quartz substrate as compared to those on the glass substrate. This difference in aggregation mass causes the polariton dephasing effect [37] in which the material response is out-of-phase with respect to the driving field. This is responsible for the optical properties of the material. The split PL peaks, called peak 1 and peak 2, in two different energy regions undergo a red shift and a blue shift, with a difference in the slope and the point where the shift changes from red to blue. The differences are related to the difference in the aggregated density and pattern as explained by the theory. Figure 4a–c shows the UV–vis absorption spectra of AuNPs in solution, deposited on quartz and deposited on glass substrate, respectively. The absorption band of AuNPs on quartz is quite broad, with a FWHM of ≈150 nm, while for the AuNPs on a glass substrate, the band is less broad (FWHM ≈100 nm), due to lower aggregation, as evidenced in the SEM images of Figure 2. The second peak for the glass substrate is due to the splitting in the plasmonic resonance due to the hybridization effect [19]. The absorption spectra of AuNPs in solution shows a peak at ≈521 nm which is much sharper (FWHM ≈40 nm) than both cases of quartz and glass substrate. This confirms that the surfactant in the colloidal solution prevents the AuNPs from aggregating [8]. The theoretical simulation of the absorption of the spherical AuNPs is finally shown in Figure 4d, where the positions of the absorbance peaks are found to be ≈525 nm in solution, ≈535 nm on quartz, and ≈540 nm on glass, for spherical AuNPs of 14 nm diameter. The theoretical curve is calculated using Mie theory based on the analytical solution of Maxwell’s equations for light scattered by spherical particles [38]. However, the theory cannot be used to calculate the scattering for different geometries; instead, a discrete dipole approximation is used for such geometries. The calculation parameters are adopted from previous publications [39] based on the analytical formula in [40]. The refractive index of the substrates were assumed to be 1.448 for quartz [40] and 1.511 for glass [41]. The difference in the measured and theoretically expected values is attributed to the geometrical factors discussed previously [18].

Figure 5 shows the shift of the PL peaks with changing excitation wavelength for AuNPs in solution, on the glass and on the quartz substrate. The data points in the figure were obtained by fitting the peaks (as shown in Supporting Information File 1 Figures S2–S4) also based on Equation 2. We named peak 1 and peak 2 the two peaks at high and low energy, respectively. The origin of the PL emission from AuNP aggregates is a matter of theoretical debate. The change in the PL peak position with changing excitation wavelength implies that the PL emission does not originate only from particle plasmon emission but also from the modulation of the interband transition d–sp [42]. The PL emission could be due to the following three steps: (i) photo-excited d-band holes that (ii) relax through nonradiative recombination to states within the d-band where momentum conservation and energy is allowed, and (iii) creation of particle plasmons, which decay by emitting a photon. In the case of aggregated systems, the appearance of the localized surface plasmon is related the fractal dimension of the aggregates. Anderson localization is responsible for enhancing the localized field, and hence, it appears in the PL emission [43]. The localized enhanced field gives rise to a PL emission in which the peak is greatly dependent on the size and shape of the AuNPs [44]. The blue shift observed in the peak position in Figure 5a–c for peak 1 and peak 2 can be attributed to a quantum size effect from the AuNPs [45]. In Figure 5b, both peak 1 and 2 show a red shift in the high energy excitation region, while in the low energy region, the shift is reverted to blue. In Figure 5c it is reversed: both peak 1 and 2 show a blue shift in the high energy region, while a red shift occurs in the low excitation energy region. The shift is totally different in case of Figure 5a. All these differences are attributed to the split of hybridized plasmon resonance and the aggregation mass as explained before.

Figure 5d shows the dependence of the emission linewidth and thus the dephasing time of the AuNPs on the surrounding medium for both solutions, on glass substrate or quartz substrate. On the high energy side of Figure 5d, a separated cluster of data points appears for glass. This is probably due to the somewhat irregular behavior of aggregation on this substrate, where sometimes the nanoparticles did not totally wet the surface and formed a tree-like network. Hence, aggregated islands appeared which had weak communication with the neighboring ones at longer wavelengths. When comparing the data in Figure 5d for the same peak from different form of aggregates, the AuNP aggregates on glass are found to have a larger dephasing time than on quartz and in solution, which implies stronger field enhancement. However, the scattered values indicate that the plasmonic response does not originate from a uniform distribution of the aggregated mass. In other words, the size distribution and the distances between the aggregated objects, following Equation 6, vary widely around those of the aggregates. We noticed that in the case of a quartz substrate, the dephasing is more regular and implies lower field confinement [46–47] than for the colloidal solution, where the AuNPs have the weakest Anderson localization. The dephasing rate is related to the time constant of the inelastic decay of the plasmon population [48–50]. The dependence of the dephasing rate on the aggregation geometry is important in sensors and in surface enhanced Raman spectroscopy applications, where the metal nanoparticles are often required to have a very slow dephasing rate [46].

However, to understand the effect of aggregation, one should think about the origin of the PL emission. In fact, optically excited metal surfaces show no PL or very little emission. In case of smooth gold surface, the PL emission has an efficiency of ≈10^−10^, where the emission follows the transition from 5d to 6sp bands. The reason for this low PL intensity is the nonradiative relaxation process of the photo-excited carriers. The Coulomb carrier–carrier scattering is known as a nonradiative process, which is much faster than radiative recombination, thus quenching the PL emission. But in the case of AuNPs with aggregates, the PL efficiencies increase up to ≈10^−4^, which is very high compared to the bulk gold surface [51]. According to Boyd et al. [42] the PL emission from the radiative recombination of SP band electrons with d-band holes is enhanced by the local field of the oscillations, which suggests speeding up the radiative recombination and subsequent radiation of the particle plasmons, giving rise to the observed PL. The lifetime of the excited particle plasmon is shown in Table 1. The exponential fits of the decaying signals pointed out the presence of two different lifetimes, to each of which a percentage can be assigned that correlates to the amount of material corresponding to the given decay time. The values show that the emission is faster in the case of quartz than on glass and in colloidal form. This can be due to the occurrence of a different distribution of hot spots where the optical field is highly localized due to the geometrical arrangement. Two suggested pathways for this geometrically based enhancement are suggested: (1) slowing down of the process of nonradiative relaxation of the excited carriers; and (2) speeding up of the process of the radiative processes in the nanoparticles as compared with bulk gold.

**Table 1:** Lifetime measurement of the PL emission from AuNPs dispersed in different media. The percent value is the probability that the given value actually represents the correct lifetime.

Sample	PL lifetime (ns)

AuNPs on glass	3.1 (55%), 0.1 (44%)
AuNPs on quartz	2.55 (67%), 0.54 (33%)
AuNPs in solution	4.0 (69%), 0.5 (31%)

One reason for the slowing down of the nonradiative relaxation of excited carriers is the size-induced opening of a gap in the density of states, which is very common in gold particles smaller than 3 nm diameter, where the density of states is far from bulk [52]. However, our AuNPs are of 14 nm average diameter. Another effective process is the carrier–phonon scattering, but this process is as fast as in the bulk, so it cannot contribute to the enhancement of the PL. Coulomb scattering is another nonradiative process, but it is faster in nanoparticles than in bulk metal because of the size-dependent screening effect, which helps in accelerating the electron–electron scattering. This is one reason for emission by hot carriers of particle plasmons, which induces emission from the collective oscillation of conduction electrons. Hence, the enhancement cannot be due to the slowing down of nonradiative relaxation [5–6] while the speeding up of the radiative process due to geometrical enhancement in the localized field could be the possible reason. Accordingly, the Anderson localization in metal nanoparticles aggregates could be used to enhance the PL emission by tuning the aggregate pattern using the surrounding medium dielectric constant.

## Conclusion

We showed that the optical properties of AuNP aggregates are very dependent on their aggregation pattern and related geometrical factors. The aggregation creates hotspots where the optical energy is localized in the plasmonic structure, known as Anderson localization. Different substrates are found to induce different aggregation density and coordination number, which is confirmed from the shift in the XPS data. The difference in aggregation induces changes in the dephasing time, linewidth and particle plasmon resonance position. The AuNPs on quartz show a dephasing which is more regular and implies lower field confinement than for AuNPs on glass that have scattered values, and the colloidal AuNPs that have the weakest localization. The difference is attributed to the difference in the geometrical arrangement, which is more ordered on quartz than on a glass substrate. The geometrical arrangement of the AuNPs is also responsible for the speeding up of the radiative process in the localized field, giving rise to an enhancement in its PL emission. The change in the PL peak position with changing excitation wavelength implies that the PL emission does not originate only from particle plasmons, but also from the modulation of the interband transition d–sp. Hence, we conclude that the modulation of the inter-subband transition has a factor dependency on geometry as well. The enhancement of the PL emission from AuNP aggregates that experience Anderson localization is due to the increase of the radiative process in the localized field.

These results suggest that by fabricating special surface patterns using electron beam lithography or by self-assembly, the localization of light can be engineered. The possibility to generate Anderson localization by control of the aggregation pattern of self-assembled nanoparticles can be an inexpensive way to engineer the PL emission for consumer optoelectronic devices.

## Experimental

### Materials

Hydrogen tetrachloroaurate trihydrate (HAuCl_4_·3H_2_O) of 99.99% purity (Alfa Aesar, Karlsruhe, Germany) and trisodium citrate (TSC) of 99.99% purity (Sigma-Aldrich, Milan, Italy) were used as received. Milli-Q water of high purity and resistivity of 18.2 MΩ·cm was used during all the AuNP synthesis, cleaning and sample preparation. Substrates of ≈4 cm^2^ surface area were cut off of ≈1 mm thick plates of either glass from commercial optical microscope slides (Menzel-Gläser, Germany) and pure fused silica (Heraeus, Germany), called quartz in the following. Before use, the substrates were cleaned by successive sonication (5 min at each step) in warm acetone, then isopropanol, then water, and finally blown dry under a nitrogen stream. As the last step, the substrates were treated for 20 min inside a UV-ozone cleaner Procleaner (BioForceNanosciences, USA).

### Synthesis and sample preparation

The synthesis of AuNPs was performed using the Frens method [53]. The TSC was employed as a reducing and surface stabilizing agent. Briefly, 10 mL of TSC (70 mM) was added with vigorous stirring to boiled HAuCl_4_·3H_2_O (100 mL, 1 mM). The reaction continued for 20 min, where the solution color changed from pale yellow to a deep pink color, indicating the formation of AuNPs. Finally, the reaction solution was cooled down to room temperature and the nanoparticles were collected by centrifugation at 10,000 rpm for 20 min. Three cycles of centrifugation were carried out in which the supernatant was replaced every time by milli-Q water. Finally, the AuNPs were collected and redispersed in milli-Q water. The obtained colloidal solution of AuNPs was then drop cast on the substrates in ambient conditions in order to obtain the films of AuNP aggregates. The substrates were cleaned by sonication for 20 min in a 1:1 mixture of ethanol/milli-Q water, prior to drop casting.

### Sample characterization

The AuNP absorbance was characterized by a spectrophotometer (Cary 6000i, Agilent, Santa Clara, CA, USA). The AuNP aggregate geometry was characterized by a field-emission scanning electron microscope (SEM) (JSM-7500F, Jeol, Tokyo, Japan). X-ray diffraction (XRD) was carried out on the samples by means of an X-Ray diffractometer (SmartLab, Rigaku, Tokyo, Japan). The PL was measured using a spectrophotometer (Fluromax-4, Horiba Jobin-Yvon, Kyoto, Japan), and the lifetime was measured using the fluorescence lifetime spectrophotometer (ChronosBH, ISS, Champaign-Urbana, IL, USA). The nanosecond time-resolved emission profiles were fitted to an exponential decay, allowing for the detection of the different lifetimes. The shift in the binding energy of Au for the core level 4f7/2 and 4f5/2 from the bulk value of the AuNP aggregates was measured by X-ray photoelectron spectroscopy (XPS) performed using an electron spectrometer (Lab2, Specs, Berlin, Germany) equipped with a monochromatic X-ray source (set at 1253 eV) and with a hemispherical energy analyzer (Phoibos, HSA3500, also from Specs). The applied voltage of the Mg Kα X-ray source was set at 10 kV and the applied current at 15 mA. The pressure in the analysis chamber was ≈2 × 10^−9^ mbar. The large area lens mode was used for both wide and narrow scans. For the wide scan, the energy pass was 90 eV, the energy step was 0.5 eV and the scan number was 2. For the narrow high-resolution scan, the energy pass was 30 eV, the energy step was 0.1 eV, and the scan number was 20. A flood gun was used to neutralize the surface charge, having an energy of 7 eV and a filament current of 2.6 A. The C1s was charge corrected to 285.0 eV.

## Supporting Information

File 1 Additional experimental information.
