# Supplementary material for: Effect of Anderson localization on light emission from gold nanoparticle aggregates
Source: Beilstein J Nanotechnol. 2016 Dec 16;7:2013–22. doi: 10.3762/bjnano.7.192 (PMC5238672; doi:10.3762/bjnano.7.192)
Supplement: File 1 — Additional experimental information. [file Beilstein_J_Nanotechnol-07-2013-s001.pdf]

Supporting Information

for

Effect of Anderson localization on light emission from gold nanoparticle aggregates

Mohamed H. Abdellatif[1], Marco Salerno[1,*,§], Gaser N. Abdelrasoul[1], Ioannis Liakos[2], Alice Scarpellini[3], Sergio Marras[3], and Alberto Diaspro[1,4]

Address: [1]Nanophysics Department, Istituto Italiano di Tecnologia, via Morego, 30, I-16163 Genova, Italy, [2]Smart Materials Group, Istituto Italiano di Tecnologia, via Morego, 30, I-16163 Genova, Italy, [3]Nanochemistry Department, Istituto Italiano di Tecnologia, via Morego, 30, I-16163 Genova, Italy and [4]Department of Physics, University of Genoa, via Dodecaneso, 33, I-16146 Genova, Italy

Email: Marco Salerno* - marco.salerno@iit.it

* Corresponding author

[§]Tel.: 0039-010-71781-444; Fax: 0039-010-720321

Additional experimental information

1. Morphology of the substrates

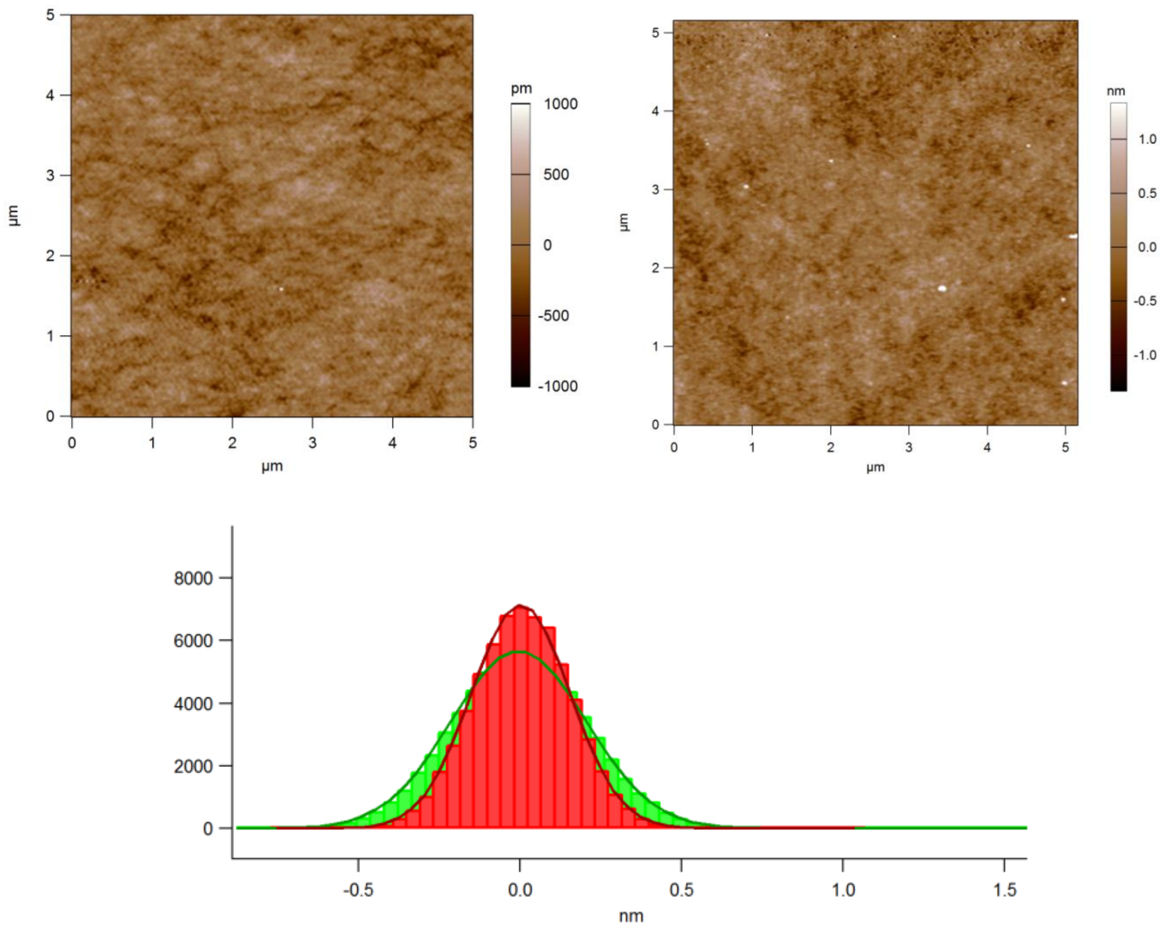

Figure S1: Quartz: left image, red distribution; glass: left image, green distribution.

Apart from occasionally overlaid nanoparticles, due to cleaning issues or environmental contaminant particles, the surfaces looked equally flat and smooth. No spatial parameters were considered in the analysis, as the substrates presented no significant in-plane texture and all the morphology could be assigned to Amplitude nature. The height distributions appeared symmetrical in both cases (low skewness and kurtosis), with Sq (RMS) of 0.15 nm and 0.20 for quartz and glass, respectively, not significantly different from each other.

2. Clarification of the PL peak fitting

Examples follow:

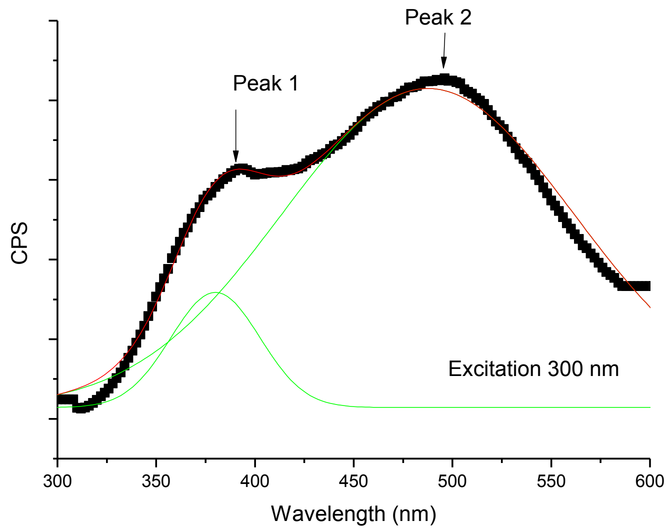

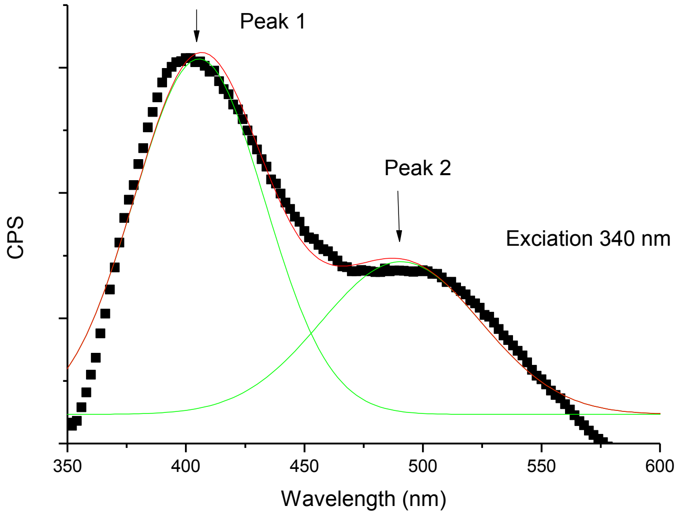

Figure S2: The PL emission indicating peak 1 and peak 2 for AuNPs / **Glass** for selected excitation at 300 nm and 340 nm.

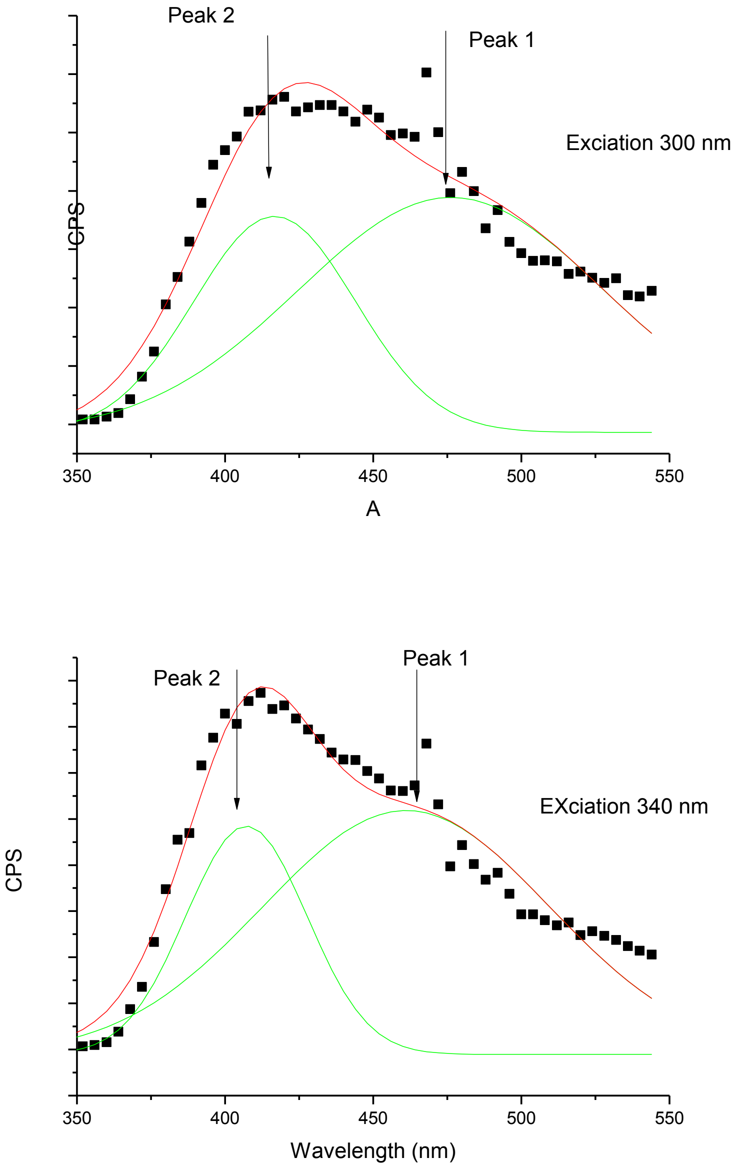

Figure S3: The PL emission indicating peak 1 and peak 2 for AuNPs / **Quartz** for selected excitation at 300 nm and 340 nm.

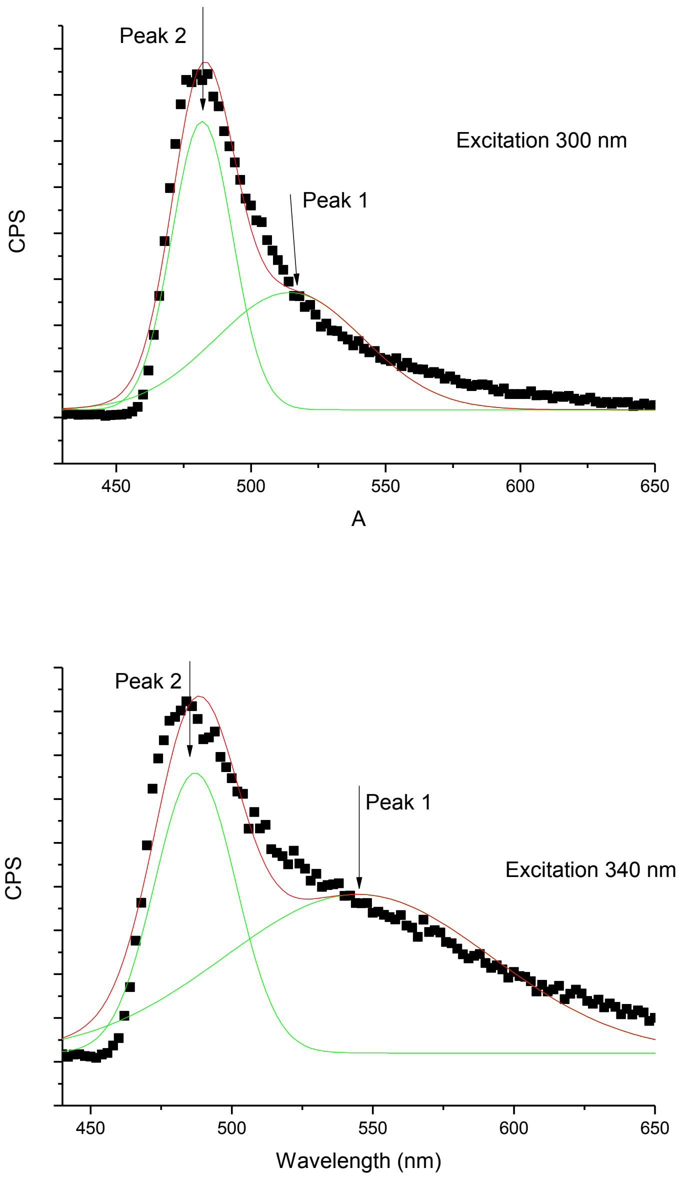

Figure S4: The PL emission indicating peak 1 and peak 2 for AuNPs / **Solution** for selected excitation at 300 nm and 340 nm.
